# Association of Net Worth and Ambulatory Blood Pressure in Early Middle-aged African American Women

**DOI:** 10.1001/jamanetworkopen.2022.0331

**Published:** 2022-02-24

**Authors:** Telisa Spikes, Raphiel Murden, Izraelle I. McKinnon, Samantha Bromfield, Miriam E. Van Dyke, Renee H. Moore, Frederic F. Rahbari-Oskoui, Arshed Quyummi, Viola Vaccarino, Tené T. Lewis

**Affiliations:** 1 School of Nursing, Emory University, Atlanta, Georgia; 2 Department of Biostatistics and Bioinformatics, Rollins School of Public Health, Emory University, Atlanta, Georgia; 3 Department of Epidemiology, Rollins School of Public Health, Emory University, Atlanta, Georgia; 4 Emory University, Atlanta, Georgia; 5 Department of Epidemiology and Biostatistics, Drexel University, Dornsife School of Public Health, Philadelphia, Pennsylvania; 6 Division of Nephrology, Department of Medicine, Emory University School of Medicine, Atlanta, Georgia; 7 Division of Cardiology, Department of Medicine, Emory University School of Medicine, Atlanta, Georgia

## Abstract

**Importance:**

Low socioeconomic status (SES) in the form of educational level and income has been linked to greater cardiovascular risk across cohorts; however, associations have been inconsistent for African American individuals. Net worth, a measure of overall assets, may be a more relevant metric, especially for African American women, because it captures longer-term financial stability and economic reserve.

**Objective:**

To examine whether net worth is associated with increased ambulatory blood pressure (ABP), a marker of cardiovascular disease (CVD) risk, independent of educational level and income, in young to middle-aged African American women.

**Design, Setting, and Participants:**

A cross-sectional, community-based study conducted in the southeastern US was performed using 48-hour ambulatory BP monitoring. Participants included 384 African American women aged 30 to 46 years without clinical CVD recruited between December 16, 2016, and March 21, 2019; data analysis was performed from September 2020 to December 2021.

**Exposures:**

Self-reported net worth (total financial assets minus debts), self-reported educational level, and self-reported income.

**Main Outcomes and Measures:**

Mean daytime and nighttime BP levels, assessed via 48-hour ABP monitoring and sustained hypertension (ABP daytime and clinic BP ≥130/80 mm Hg).

**Results:**

The 384 African American women in this study represented a range of SES backgrounds; mean (SD) age was 38.0 (4.3) years. Excluding 66 women who were not receiving antihypertensive medications, in linear regression models adjusted for age, marital status, educational level, family income, and family size, women reporting a negative net worth (debt) had higher levels of daytime (β = 6.7; SE = 1.5; *P* < .001) and nighttime (β = 6.4; SE = 1.4; *P* < .001) systolic BP, compared with women reporting a positive net worth. Similar associations were observed with sustained hypertension: women reporting a negative net worth had 150% higher odds (odds ratio, 2.5; 95% CI, 1.3-4.7) of sustained hypertension than those reporting a positive net worth. Associations remained significant after additional adjustments for smoking, body mass index, psychosocial stress due to debt, and depressive symptoms and were similar, although attenuated, when women receiving antihypertensive medications were included and treatment was controlled for in all analyses.

**Conclusions and Relevance:**

In this cross-sectional study, having a negative net worth (ie, debt) was associated with elevated BP in African American women, independent of traditional indicators of SES. This finding suggests that limited assets or a lack of economic reserve may be associated with poor CVD outcomes in this at-risk group.

## Key Points

**Question:**

Is net worth (ie, assets or economic reserve) associated with blood pressure (BP) in African American women, independent of educational level and income?

**Findings:**

In this cross-sectional study, a socioeconomically diverse cohort of 384 African American women aged 30 to 46 years undergoing 48-hour BP monitoring, a negative (ie, in debt) vs positive net worth was associated with increased daytime and nighttime BP and higher odds of sustained hypertension. Findings were independent of risk factors, including educational level and income.

**Meaning:**

This study suggests that net worth may be a greater risk factor for increased BP than traditional socioeconomic indicators in African American women.

## Teaser

This cross-sectional study examines ambulatory blood pressure levels in African American women of varying net worth status.

## Introduction

Evidence has documented associations between low socioeconomic status (SES), traditionally measured at the individual level by educational level and/or income, and greater cardiovascular disease (CVD) morbidity and mortality.^1,2^ However in the US, these associations have been less consistently observed for African American individuals relative to White individuals.^3,4,5^ Some have argued that this difference is primarily owing to the inability of traditional SES measures to adequately capture resources, such as wealth and/or assets, that are limited among the African American population, largely due to historical policies stemming from racism.^6,7,8^ Differential access to US Government Issue bill benefits,^9^ redlining (linking risk to residential maps based on racial composition),^10,11^ and deed covenants (preventing the sale of homes in some areas to African American individuals)^10,11^ constrained access to homeownership and home equity accrual in high-value areas, which, in addition to laws impeding enrollment in many colleges and universities across the US through the 1960s and early 1970s, directly affected opportunities for African American individuals to achieve upward mobility and attain generational wealth.^6,12^ The legacy of these historical policies has ongoing importance, as recent data found that on average, African American households had $46 600 in total assets, compared with $264 700 in White households.^13^

Although the connection between traditional SES indicators and CVD is well studied, the association between the presence or absence of assets and CVD risk is relatively underexplored, especially among the African American population.^14^ Thus, the current analysis examined associations between net worth, a measure of wealth, defined as the total value of all accumulated assets minus any liabilities or debts,^15^ and elevated blood pressure (BP), a predictor of CVD,^16,17^ in a cohort of young to middle-aged African American women. We focused on African American women because studies report that, compared with White men, White women, and African American men, African American women are the most affected by the wage gap,^18,19^ the most likely to be single parents/heads of households,^19,20^ and among college-educated adults, the most burdened by student loan debt.^21^ Consequently, African American women may be uniquely vulnerable to the association between limited assets (ie, debt) and CVD risk.

In addition, there is evidence that African American women aged 35 to 44 years have rates of CVD that exceed those of similarly aged White men, White women, and African American men; thus, examining factors that might contribute to increased risk among African American women specifically is of particular importance.^22,23^ We hypothesized that, compared with African American women reporting a positive or neutral net worth, those reporting a negative net worth (ie, debt) would have higher BP measured via 48-hour ambulatory BP (ABP) monitoring, even after adjusting for traditional SES indicators and other relevant risk factors. Given prior associations between psychosocial factors and ABP,^24^ we further examined whether any observed net worth and ABP associations were independent of debt stress (ie, psychological stress surrounding negative assets) and depressive symptoms.^25^ We used ABP because of its reported associations with mortality and adverse cardiovascular outcomes over and above office BP,^26^ especially among women.^27^

## Methods

### Participants

Participants were 384 African American women enrolled in the Mechanisms Underlying the Impact of Stress and Emotions on African American Women’s Health cohort (eMethods in the Supplement). All procedures were approved by the Emory University Institutional Review Board, and all participants provided written informed consent. All participants were financially compensated for the 3-day study. This study followed the Strengthening the Reporting of Observational Studies in Epidemiology (STROBE) reporting guideline for cross-sectional studies.^28^

Women were recruited between December 16, 2016, and March 21, 2019, using residential lists from a wide range of census tracts in the greater metropolitan Atlanta, Georgia, area. Eligibility criteria included self-identification as a Black or African American woman, aged 30 to 45 years at screening, and premenopausal with 1 or both ovaries. Ineligibility criteria were pregnancy or lactation, clinical CVD history (eg, myocardial infarction, angina, and cerebral ischemia or revascularization), illnesses that influence CVD (eg, autoimmune disease, HIV/AIDS, kidney disease), psychiatric disorder treatment, illicit drug use, or shift-working (owing to alterations in circadian rhythms). Because prior studies have found that African American women do not receive substantive health gains from higher vs lower SES,^5,29,30^ by design, 50% of the participants were above and 50% were below the median income of $50 000 in Georgia at the time of recruitment. This categorization was done to more adequately understand determinants of risk across the SES spectrum in this group.

Participants completed an initial in-person visit in which height, weight, 2 seated measures of resting BP, and additional clinical data were assessed. Face-to-face interviews were conducted to obtain detailed demographic and psychosocial characteristics. Following this procedure, 48-hour ABP monitoring was conducted and participants were instructed to try their best to obtain an 80% completion rate and contact study staff with any questions or difficulties.

### ABP Monitoring

Ambulatory BP monitors (OnTrak model 90227; Spacelabs Healthcare) were used to obtain ABP readings over 2 consecutive days. Participants were trained on proper application and removal techniques and instructed to remove the device only to shower or bathe. Prior to fitting, the ABP monitoring was programmed to record systolic BP (SBP) and diastolic BP (DBP) every 30 minutes during the day (8 am to 10 pm) and every hour during the night. On completion, the ABP monitoring device was returned to study staff. Readings were downloaded with Sentinel Software, version 10.5, from Spacelabs Healthcare.

### Net Worth

As in prior research,^31,32^ net worth was assessed with a single item: “Suppose you and others in your household were to sell all of your major possessions (including your home), turn all of your investments and other assets into cash, and pay off all of your debts. Would you have something left over, break even, or be in debt?” A 3-category measure was constructed that included (1) positive net worth (left over, ie, assets exceed debt), (2) neutral net worth (break even, ie, an equal debt to assets ratio), and (3) negative net worth (in debt, ie, debts exceed assets).

### ABP Outcomes

Forty-eight hour ABP completion rates ranged from 9% to 150% (some participants wore the ABP cuff for a few hours into the next day), with 88% of women achieving a completion rate of 80%. Outcome variables were the mean of all SBP and DBP readings obtained across the 48-hour period, separated into daytime and nighttime values, and sustained hypertension according to the 2017 American College of Cardiology/American Heart Association BP guideline thresholds.^33,34^ Thus, there were 4 continuous BP outcomes (daytime SBP, nighttime SBP, daytime DBP, and nighttime DBP) and a measure of clinically increased BP, defined as an average BP 130/80 mm/Hg or higher in both clinic and daily life (eg, ABP).^33,34^ This level is in contrast to clinic BP alone, which includes some proportion of individuals who display increased BP in the clinic, but not daily (ie, white coat hypertension).

### Covariates

Covariates were chosen based on prior studies.^31,32,35,36,37,38,39^ Sociodemographic characteristics, including age, marital status (ie, married/living with partner vs unmarried/not living with partner), educational level (≤high school, some college, and ≥ college), annual family income (<$35 000, $35 000-$49 999, $50 000-$74 999, and ≥$75 000), and family size (number of individuals in the household, as an adjustment for family income only) were self-reported. Body mass index (BMI) was calculated as weight in kilograms divided by height in meters squared and modeled continuously. Current smoking and use of antihypertensives were self-reported and modeled as yes or no. As in earlier studies,^25,40^ debt stress was assessed via a 4-item index (eg, How much stress does the total debt you [and your spouse/partner] are carrying cause to you? How concerned are you that you [and your spouse/partner] will never be able to pay off these debts?). Responses were scored on a 0- to 4-point scale and summed. Possible scores ranged from 0 to 16, with higher scores indicative of more stress and worry due to debt. Cronbach α level for the debt stress scale in this cohort showed high consistency (α = .84), and scores were modeled continuously. Depressive symptoms were measured with the widely used and previously validated 21-item Beck Depression Inventory^41^ and modeled continuously.

### Statistical Analysis

Data analysis was performed from September 2020 to December 2021. Descriptive statistics were used to characterize study participants. Differences by net worth were examined using analysis of variance or χ^2^ tests. Bivariate associations among net worth and SES variables were tested using polychoric correlations for ordered data,^42^ and associations between SES variables and BP outcomes were tested with analysis of variance, χ^2^ tests, or logistic regression analyses for sustained hypertension. Multivariable linear regression models were conducted to test associations with continuous ABP outcomes. Model 0 was age adjusted only to examine associations before adjusting for other SES indicators. Model 1 was adjusted for age, marital status, educational level, income, and family size for family income. Model 2 added 1 term for BMI and smoking; model 3 adjusted for debt stress, and model 4 adjusted for depressive symptoms. This sequence was also used to examine sustained hypertension, using logistic regression analyses. Given potential correlations between net worth, educational level, and income, we also tested for multicollinearity among these variables. The variance information factors were 1.45 to 1.47 for income, 1.35 to 1.37 for educational level, and 1.13 to 1.16 for net worth, indicating a lack of multicollinearity.^43^ Initial models excluded 66 women receiving antihypertensive medicine; thus, we ran a series of secondary models including these women, adjusting for antihypertensive use as in other ABP analyses.^36,44^ All analyses were conducted in SAS, version 9.4 (SAS Institute Inc). A 2-sided value of *P* < .05 was considered statistically significant.

## Results

### Participant Characteristics

As reported in Table 1, participants’ mean (SD) age was 38.0 (4.3) (range, 30-46) years and they were from a range of educational level and income backgrounds. Positive net worth was reported by 183 women (47.7%), neutral by 89 women (23.2%), and negative by 112 women (29.2%). Women reporting positive vs negative net worth had higher incomes (eg, ≥$75 000: 86 [47.0%] vs 18 [16.1%]), were more likely to be college graduates (107 [58.5%] vs 47 [42.0%]), more likely to be married/living with a partner (84 [45.9%] vs 31 [27.7%]), more likely to have a lower BMI (mean [SD], 31.7 [7.5] vs 34.3 [8.8]), less likely to smoke (11 [6.0%] vs 18 [16.1%]), less likely to report debt stress (mean [SD], 3.6 [2.6] vs 5.6 [2.8]), and less likely to have depressive symptoms (mean [SD], 4.4 [5.1] vs 7.6 [8.1]) (Table 1). Women with a positive vs negative net worth also had lower daytime SBP values (119.0 [11.7] vs 124.4 [11.8]), lower nighttime SBP values (109.3 [11.1] vs 114.8 [11.9], lower daytime DBP values (76.2 [8.4] vs 79.2 [8.4]), lower nighttime DBP values (67.3 [8.2] vs 70.9 [8.7]), and a lower prevalence of sustained hypertension (45 [24.6%] vs 45 [40.2%]).

**Table 1.** Participant Characteristics by Net Worth Status

Characteristic	No. (%)				*P* value
	Overall	Positive	Neutral	Negative	
No. (%)	384	183 (47.7)	89 (23.2)	112 (29.2)	
Age, mean (SD), y	38.0 (4.3)	38.6 (4.4)	37.5 (4.2)	37.5 (4.1)	.049
Educational level					
≤High school	112 (29.2)	38 (20.8)	31 (34.8)	43 (38.4)	.003
Some college	83 (21.6)	38 (20.8)	23 (25.8)	22 (19.6)	
≥College	189 (49.2)	107 (58.5)	35 (39.3)	47 (42.0)	
Income, $					
<35 000	94 (24.5)	23 (12.6)	29 (32.6)	42 (37.5)	<.001
35 000-49 999	80 (20.8)	34 (18.6)	17 (19.1)	29 (25.9)	
50 000-74 999	86 (22.4)	40 (21.9)	23 (25.8)	23 (20.5)	
≥75 000	124 (32.3)	86 (47.0)	20 (22.5)	18 (16.1)	
Married/live-in partner	148 (38.5)	84 (45.9)	33 (37.1)	31 (27.7)	.007
Current smoker	37 (9.6)	11 (6.0)	8 (9.0)	18 (16.1)	.02
BMI, mean (SD)	32.8 (8.08)	31.7 (7.52)	33.01 (8.04)	34.3 (8.76)	.02
Ambulatory blood pressure, mean (SD), mm Hg					
Daytime SBP	121.3 (12.3)	119.0 (11.7)	122.2 (13.1)	124.4 (11.8)	<.001
Nighttime SBP	111.3 (11.7)	109.3 (11.1)	110.9 (11.8)	114.8 (11.9)	<.001
Daytime DBP	77.6 (8.8)	76.2 (8.4)	78.3 (9.6)	79.2 (8.4)	.01
Nighttime DBP	68.6 (8.6)	67.3 (8.2)	68.3 (9.0)	70.9 (8.7)	.002
Sustained hypertension	121 (31.5)	45 (24.6)	31 (34.8)	45 (40.2)	.02
Antihypertensive use	66 (17.2)	30 (16.4)	16 (18.0)	20 (17.9)	.93
Debt stress, mean (SD)	4.4 (2.8)	3.6 (2.6)	4.8 (2.9)	5.6 (2.8)	<.001
Depressive symptoms, mean (SD)	5.8 (6.7)	4.4 (5.1)	6.3 (6.9)	7.6 (8.1)	<.001

Abbreviations: BMI, body mass index (calculated as weight in kilograms divided by height in meters squared); DBP, diastolic blood pressure; SBP, systolic blood pressure.

### Basic Associations Among Net Worth, Traditional SES Measures, and ABP Outcomes

Net worth was significantly associated with educational level (*r* = 0.24; *P* < .001) and income (*r* = 0.41; *P* < .001). Similarly, educational level and income were significantly associated with one another (*r* = 0.48; *P* < .001). Educational level was not significantly associated with daytime SBP, with mean (SD) levels of 118.8 (10.6) mm Hg for college degree or higher, 121.07 (11.6) mm Hg for some college, and 120.0 (12.49) mm Hg for high school or less (*P* = .37). Educational level was also not associated with nighttime SBP (108.89 [9.77] mm Hg for college or higher, 110.54 [10.31] mm Hg for some college, and 110.04 [12.69] mm Hg for high school or less; *P* = .52), daytime DBP (76.25 [7.62] mm Hg for college or higher, 77.21 [8.68] mm Hg for some college, and 76.27 [9.11] mm Hg for high school or less; *P* = .72), nighttime DBP (67.36 [7.48] mm Hg for college or higher, 68.25 [8.53] mm Hg for some college, and 67.13 [9.07] mm Hg for high school or less; *P* = .69), or the prevalence of sustained hypertension (44.4% [36%] for college or higher, 23.5%[19%] for some college, and 32.1% [26%] for high school or less; *P* = .32).

Similar nonsignificant results were observed for income adjusted for family size (*P* = .34), with mean (SD) daytime SBP levels of 119.5 (11.4) mm Hg for $75 000 or more, 118.27 (10.9) mm Hg for $50 000 to $74 999, 119.32 (10.47) mm Hg for $35 000 to $49 999, and 121.29 (13.07) mm Hg for less than $35 000, with close to identical BPs between the $35 000 through $75 000 or greater range. Income was also not associated with nighttime SBP (109.44 [9.88] mm Hg for $75 000 or more, 107.74 [9.90] mm Hg for $50 000-$74 999, 109.92 [11.00] mm Hg for $35 000-$49 999, and 111.18 [12.61] mm Hg for less than $35 000; *P* = .20), daytime DBP (76.79 [10.40] mm Hg for $75 000 or more, 76.38 [7.03] mm Hg for $50 000-$74 999, 75.89 [7.47] mm Hg for $35 000-$49 999, and 76.63 [8.00] mm Hg for less than $35 000; *P* = .85), nighttime DBP (67.37 [7.59] mm Hg for $75 000 or more, 66.33 [7.10] mm Hg for $50 000-$74 999, 67.84 [7.41] mm Hg for $35 000 to $49 999, and 68.40 [10.42] mm Hg for less than $35 000; *P* = .38), or sustained hypertension (odds ratio [OR], 1.60; 95% CI, 0.81-3.15 for the $50 000 to $74 999 group; OR, 1.36; 95% CI, 6.8-2.75 for the $35 000 to $50 000 group; and OR, 0.94; 95% CI, 0.45-1.94 for the less than $35 000, with the $75 000 or more group as the referent).

### Net Worth Status and Continuous ABP Outcomes

In age-adjusted models (model 0), compared with women with a positive net worth, women with a negative (debt) or neutral net worth had higher levels of daytime SBP (β = 6.6; SE = 1.5; *P* < .001 for negative; β = 3.2; SE = 1.6; *P* = .04 for neutral). This dose-response association is illustrated in the Figure. Negative, compared with positive, net worth associations remained after adjusting for marital status and traditional SES measures (β = 6.7; SE = 1.5; *P* < .001) (Table 2), as well as smoking and BMI (β = 6.2; SE = 1.5; *P* < .001) but were attenuated slightly after adjusting for debt stress (β = 5.3; SE = 1.6; *P* = .001) and depressive symptoms (β = 5.2; SE = 1.6; *P* = .001) (Table 2).

**Figure.**
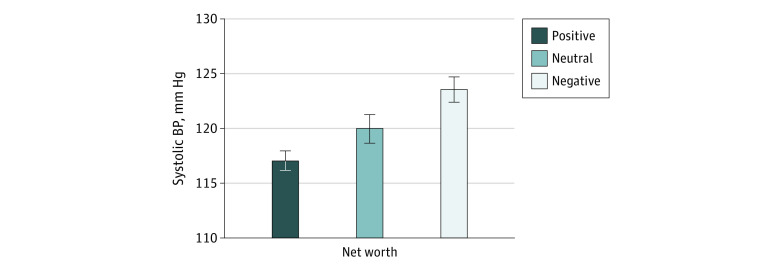
Net Worth and Daytime Systolic Blood Pressure (BP) in African American Women Values are means; error bars indicate SD *P* < .001 for overall association.

**Table 2.** Net Worth and 48-Hour Daytime Systolic Blood Pressure Among African American Women

Variable	Model 1^a^		Model 2^b^		Model 3^c^		Model 4^d^	
	β (SE)	*P* value	β (SE)	*P* value	β (SE)	*P* value	β (SE)	*P* value
Net worth^e^								
Positive	1 [Reference]		1 [Reference]		1 [Reference]		1 [Reference]	
Negative	6.7 (1.5)	<.001	6.2 (1.5)	<.001	5.3 (1.6)	.001	5.2 (1.6)	.001
Neutral	3.1 (1.6)	.06	2.9 (1.6)	.08	2.4 (1.6)	.14	2.4 (1.6)	.14
Educational level								
≥College	1 [Reference]		1 [Reference]		1 [Reference]		1 [Reference]	
≤High school	1.6 (1.7)	.36	1.1 (1.7)	.53	1.3 (1.7)	.43	1.1 (1.7)	.52
Some college	2.4 (1.7)	.17	2.3 (1.7)	.18	2.3 (1.7)	.18	2.0 (1.7)	.25
Income, $								
≥75 000	1 [Reference]		1 [Reference]		1 [Reference]		1 [Reference]	
<35 000	–0.3 (2.1)	.90	–0.4 (2.2)	.86	–0.4 (2.2)	.83	–0.7 (2.2)	.76
35 000-49 999	–1.6 (1.9)	.41	–1.4 (1.9)	.44	–1.6 (1.9)	.37	–1.7 (1.8)	.35
50 000-74 999	–2.7 (1.8)	.13	–2.7 (1.8)	.13	–2.6 (1.8)	.13	–2.8 (1.7)	.11

^a^ In addition to educational level and income, adjusted for age, marital status, and family size.

^b^ Model 1 factors plus body mass index and smoking.

^c^ Model 2 factors plus debt stress.

^d^ Model 3 factors plus depressive symptoms.

^e^ Positive, assets exceed debt; neutral, debt equal to assets; negative, debts exceed assets.

Associations were similar with nighttime SBP (eFigure in the Supplement). In age-adjusted analyses, negative, compared with positive, net worth was associated with higher nighttime SBP (β = 6.4; SE = 1.4; *P* < .001), with no significant differences between neutral vs positive net worth (β = 2.0; SE = 1.5; *P* = .20). Associations persisted in models 1 to 4 (Table 3). Results for daytime and nighttime DBP (eTable 1 and eTable 2 in the Supplement) were comparable to SBP results.

**Table 3.** Net Worth and 48-Hour Nighttime Systolic Blood Pressure

Variable	Model 1^a^		Model 2^b^		Model 3^c^		Model 4^d^	
	β (SE*)*	*P* value	β (SE)	*P* value	β (SE)	*P* value	β (SE)	*P* value
Net worth^e^								
Positive	1 [Reference]		1 [Reference]		1 [Reference]		1 [Reference]	
Negative	6.4 (1.4)	<.001	5.7 (1.4)	<.001	4.9 (1.5)	.001	4.9 (1.5)	.001
Neutral	2.0 (1.5)	.20	1.7 (1.5)	.27	1.2 (1.5)	.41	1.3 (1.5)	.40
Educational level								
≥College	1 [Reference]		1 [Reference]		1 [Reference]		1 [Reference]	
≤High school	1.3 (1.6)	.43	0.6 (1.6)	.73	0.8 (1.6)	.61	0.6 (1.6)	.70
Some college	1.7 (1.6)	.30	1.6 (1.6)	.33	1.5 (1.6)	.34	1.4 (1.6)	.41
Income, $								
≥75 000	1 [Reference]		1 [Reference]		1 [Reference]		1 [Reference]	
<35 000	–0.7 (2.0)	.72	–0.9 (2.0)	.63	–1.0 (2.0)	.61	–1.2 (2.0)	.55
35 000-49 999	–1.2 (1.8)	.51	–1.1 (1.7)	.54	–1.3 (1.7)	.47	–1.4 (1.7)	.45
50 000-74 999	–3.2 (1.7)	.05	–3.2 (1.6)	.05	–3.2 (1.6)	.05	–3.3 (1.6)	.04

^a^ In addition to educational level and income, adjusted for age, marital status, and family size.

^b^ Model 1 factors plus body mass index and smoking.

^c^ Model 2 factors plus debt stress.

^d^ Model 3 factors plus depressive symptoms.

^e^ Positive, assets exceed debt; neutral, debt equal to assets; negative, debts exceed assets.

### Net Worth Status and Sustained Hypertension

In age-adjusted analyses, women reporting a negative net worth had 150% higher odds of sustained hypertension (OR, 2.5; 95% CI, 1.3-4.7) than their counterparts reporting a positive net worth. There were no associations observed between women reporting a neutral (OR, 1.7; 95% CI, 0.9-3.4) compared with positive net worth. Findings were similar after adjusting for smoking and BMI (OR, 2.5; 95% CI, 1.3-4.7) and were slightly, but not significantly, attenuated after adjusting for debt stress (OR, 2.1; 95% CI, 1.1-4.2) and depressive symptoms (OR, 2.1; 95% CI, 1.1-4.2) (Table 4).

**Table 4.** Net Worth and Sustained Hypertension

Variable	Odds ratio (95% CI)			
	Model 1^a^	Model 2^b^	Model 3^c^	Model 4^d^
Net worth^e^				
Positive	1 [Reference]	1 [Reference]	1 [Reference]	1 [Reference]
Negative	2.5 (1.3-4.7)	2.5 (1.3-4.7)	2.1 (1.1-4.2)	2.1 (1.1-4.2)
Neutral	1.7 (0.9-3.4)	1.7 (0.8-3.3)	1.5 (0.8-3.1)	1.5 (0.7-3.2)
Educational level				
≥College	1 [Reference]	1 [Reference]	1 [Reference]	1 [Reference]
≤High school	1.4 (0.7-2.9)	1.5 (0.7-3.1)	1.6 (0.7-3.3)	1.5 (0.7-3.2)
Some college	1.5 (0.8-3.1)	1.6 (0.8-3.3)	1.6 (0.8-3.3)	1.6 (0.8-3.2)
Income, $				
≥75 000	1 [Reference]	1 [Reference]	1 [Reference]	1 [Reference]
<35 000	1.2 (0.5-3.0)	1.3 (0.5-3.2)	1.3 (0.5-3.2)	1.3 (0.5-3.1)
35 000-49 999	1.2 (0.5-2.5)	1.2 (0.5-2.7)	1.2 (0.5-2.6)	1.2 (0.5-2.6)
50 000-74 999	1.0 (0.4-1.8)	0.8 (0.4-1.8)	0.8 (0.4-1.8)	0.8 (0.4-1.8)

^a^ In addition to educational level and income, adjusted for age, marital status, and family size.

^b^ Model 1 factors plus body mass index and smoking.

^c^ Model 2 factors plus debt stress.

^d^ Model 3 factors plus depressive symptoms.

^e^ Positive, assets exceed debt; neutral, debt equal to assets; negative, debts exceed assets.

### Secondary and Exploratory Analyses

Findings for continuous ABP outcomes and sustained hypertension were similar, although slightly attenuated in models including 66 women receiving antihypertensive medication (eTables 3-7 in the Supplement). Exploratory analyses testing interactions between net worth and educational level, debt stress, and depressive symptoms yielded null results (eResults in the Supplement).

## Discussion

To our knowledge, this is the first study to examine associations between a measure of wealth, net worth, and CVD risk in a cohort of African American individuals, with a focus on African American women. In this socioeconomically diverse cohort of young to middle-aged African American women, we observed an association between net worth and 48-hour ABP, with African American women who reported that they would be in debt having daytime SBP levels that were approximately 6.7 mm Hg higher than women who would have something left over, even after adjusting for sociodemographic and behavioral covariates, remaining significant after further adjustments for BMI and smoking. Similar associations were observed for sustained hypertension, with African American women reporting debt having 150% higher odds of sustained hypertension, compared with those reporting having something left over. Associations were attenuated but remained after adjusting for debt stress and depressive symptoms. Consistent with prior studies of African American individuals, we did not observe associations between educational level or income and BP outcomes.

Our finding that net worth was associated with ABP outcomes even after adjusting for educational level and income suggests that net worth as an index of SES may have an association with CVD risk—and potentially outcomes—that is not explained by traditional SES measures alone, especially among African American women. Net worth is the result of accumulated assets and differs substantially between African American and White individuals owing to historical policies, resulting in a relative wealth disadvantage for African American individuals that begins in childhood and widens over the life course.^11,45,46,47^ Thus, in addition to their lower likelihood of inheriting wealth because of their race, as a result of the aforementioned structural factors related to both race and gender (eg, the wage gap,^18,19^ lower likelihood of dual-earner partnerships,^18,19,48^ and high rates of single-parenthood),^19,20^ and occupational sorting into lower-wage service positions even when college educated,^49^ African American women may also be less likely to attain wealth than individuals from other race and gender groups.

Our observations in African American women differ from earlier work in a predominantly White cohort of young adults. Sweet et al^32^ found no association between self-reported debt-to-asset (debt) ratio and SBP, but observed a significant association with DBP. In this cohort, women with negative net worth (ie, those reporting debt) had higher BPs compared with those reporting positive net worth across all ABP outcomes. Possible reasons for the differences include our focus on ambulatory rather than resting BP, as well as our focus on African American women, who may be more affected by a lack of assets, given their high level of economic vulnerability relative to other race-gender groups.

Previous studies have documented associations between negative net worth and stress, anxiety, and depression in predominantly White cohorts, and negative net worth was associated with higher levels of debt stress and depressive symptoms in our sample.^31,32,37^ However, although our observed associations between negative net worth/debt and BP outcomes were attenuated after adjusting for debt stress and depressive symptoms, they were not eliminated. We also tested whether debt stress or depressive symptoms amplified the association between net worth and BP outcomes in exploratory analyses; these interactions were nonsignificant, suggesting that other pathways likely play a role.

Net worth and assets are major contributors to home ownership in high-equity neighborhoods, and neighborhood poverty and other markers of adverse residential environments are known contributors to excess CVD risk.^1^ Assets also provide economic security that can protect individual households from economic shocks.^45^ Studies of African American women have found that negative financial events, such as job losses and recessions, have a deleterious association with physiological aging,^50^ and although we do not have data on negative financial events in the years preceding our data collection, numerous studies have documented the particularly deleterious influence of the 2009 Great Recession on African American individuals at all SES levels who lost more wealth and experienced a markedly slower economic recovery in the decade following the recession (2009-2019) relative to other racial and ethnic groups.^49,51^ Thus, it is possible that the African American women in this cohort who reported being in debt experienced serious financial setbacks in the years before our assessment from which participants who reported having something left over were buffered. It is also conceivable that assets provide individuals with a sense of safety and security in everyday life, in contrast to debt, which could promote a sense of vigilance around financial matters (independent of debt stress and depression) that might, in turn, increase physiological arousal and increased BP.^52^ Future studies that identify the range of psychological and physiological mechanisms through which debt might affect BP are needed.

### Limitations

This study has limitations. The observed findings are cross-sectional; thus, causality cannot be established. Furthermore, net worth was self-reported and could have been subject to social desirability or recall bias. In addition, this cohort was limited to African American women in a southeastern city, and, although these results extend previous findings in White populations to a group that is at greater risk of both high levels of indebtedness and increased BP, results may not generalize to other race-gender groups or individuals from other geographic regions. Furthermore, we focused on early midlife, because previous studies have reported that this is a life stage when increases in BP are steepest for African American women compared with other race-gender groups,^53,54^ and more recent research has noted that women experience CVD events at lower thresholds than men, with associations particularly pronounced in those younger than 52 years.^55^ Nonetheless, it is unclear whether findings would be similar in younger or older age groups. In addition, this cohort featured African American women at high and low SES levels by design; thus, college-educated women were overrepresented in this cohort (50% compared with 35.9% nationally).^56^ However, this group is typically underrepresented in CVD research, and our findings suggest that their inclusion offers some insight into the relative importance of net worth and assets, even in the context of a favorable profile on traditional indicators of SES that should be protective against CVD risk.

## Conclusions

The findings of this study suggest that having a negative net worth may be associated with BP outcomes in young to middle-aged African American women. Although further studies are needed to identify potential mechanisms that might explain the observed results, these findings have particular salience in the context of the COVID-19 pandemic, because job loss in 2020 disproportionately affected women, specifically those from racial and ethnic minority backgrounds.^57^ Furthermore, relative to all other race-gender groups, African American women experienced the slowest recovery in employment through November 2021.^58^ However, additional research is needed to document the longitudinal outcomes of the COVID-19 pandemic and resulting economic recession on net worth and CVD risk and outcomes in African American women, as well as other race-gender groups. Policies that reduce the enduring racial wealth gap could be explored as potential structural-level interventions^45,46^ might ultimately reduce CVD risk in African American women.

## Notes

Supplement. **eMethods.** Differences Between Women Included Versus Excluded From the Current Analysis **eResults.** Exploratory Analyses **eFigure.** Net Worth and Nighttime Systolic Blood Pressure in African American Women **eTable 1.** Net Worth and 48-Hour Daytime Diastolic Blood Pressure Among African American Women Not Taking Antihypertensive Medications **eTable 2.** Net Worth and 48-Hour Nighttime Diastolic Blood Pressure Among African American Women Not Taking Antihypertensive Medications **eTable 3.** Net Worth and 48-Hour Daytime Systolic Blood Pressure Among African American Women, Including Those on Antihypertensive Medications **eTable 4.** Net Worth and 48-Hour Nighttime Systolic Blood Pressure Among African American Women, Including Those on Antihypertensive Medications **eTable 5.** Net Worth and 48-Hour Daytime Diastolic Blood Pressure Among African American Women, Including Those on Antihypertensive Medications **eTable 6.** Net Worth and 48-Hour Nighttime Diastolic Blood Pressure Among African American Women, Including Those on Antihypertensive Medications **eTable 7.** Net Worth and Sustained Hypertension Among African American Women, Including Those on Antihypertensive Medications
