# Supplementary material for: Association of Net Worth and Ambulatory Blood Pressure in Early Middle-aged African American Women
Source: JAMA Netw Open. 2022 Feb 24;5(2):e220331. doi: 10.1001/jamanetworkopen.2022.0331 (PMC8874347; doi:10.1001/jamanetworkopen.2022.0331)
Supplement: Supplement. — eMethods. Differences Between Women Included Versus Excluded From the Current Analysis eResults. Exploratory Analyses eFigure. Net Worth and Nighttime Systolic Blood Pressure in African American Women eTable 1. Net Worth and 48-Hour Daytime Diastolic Blood Pressure Among African American Women Not Taking Antihypertensive Medications eTable 2. Net Worth and 48-Hour Nighttime Diastolic Blood Pressure Among African American Women Not Taking Antihypertensive Medications eTable 3. Net Worth and 48-Hour Daytime Systolic Blood Pressure Among African American Women, Including Those on Antihypertensive Medications eTable 4. Net Worth and 48-Hour Nighttime Systolic Blood Pressure Among African American Women, Including Those on Antihypertensive Medications eTable 5. Net Worth and 48-Hour Daytime Diastolic Blood Pressure Among African American Women, Including Those on Antihypertensive Medications eTable 6. Net Worth and 48-Hour Nighttime Diastolic Blood Pressure Among African American Women, Including Those on Antihypertensive Medications eTable 7. Net Worth and Sustained Hypertension Among African American Women, Including Those on Antihypertensive Medications [file jamanetwopen-e220331-s001.pdf]

Supplementary Online Content

Spikes T, Murden R, McKinnon II, et al. Association of net worth and ambulatory blood pressure in early middle-aged African American women. *JAMA Netw Open*. 2022;5(2):e220331. doi:10.1001/jamanetworkopen.2022.0331

eMethods. Differences Between Women Included Versus Excluded From the Current Analysis

eResults. Exploratory Analyses

eFigure. Net Worth and Nighttime Systolic Blood Pressure in African American Women

eTable 1. Net Worth and 48-Hour Daytime Diastolic Blood Pressure Among African American Women Not Taking Antihypertensive Medications

eTable 2. Net Worth and 48-Hour Nighttime Diastolic Blood Pressure Among African American Women Not Taking Antihypertensive Medications

eTable 3. Net Worth and 48-Hour Daytime Systolic Blood Pressure Among African American Women, Including Those on Antihypertensive Medications

eTable 4. Net Worth and 48-Hour Daytime Systolic Blood Pressure Among African American Women, Including Those on Antihypertensive Medications

eTable 5. Net Worth and 48-Hour Daytime Diastolic Blood Pressure Among African American Women, Including Those on Antihypertensive Medications

eTable 6. Net Worth and 48-Hour Nighttime Diastolic Blood Pressure Among African American Women, Including Those on Antihypertensive Medications

eTable 7. Net Worth and Sustained Hypertension Among African American Women, Including Those on Antihypertensive Medications

This supplementary material has been provided by the authors to give readers additional information about their work.

© 2022 Spikes T et al. *JAMA Network Open.*

eMethods

Differences between Women included versus excluded from the Current Analysis

Of the N=414 women who participated in the ABPM assessment, 18 were excluded because they volunteered "I don't know," re: their net worth. These women were less likely to earn over $75,000, be college educated, or be married/living with a partner than women who knew their net worth (all p-values<.05), but did not differ on ABP outcomes, behavioral, or psychosocial factors. Of the remaining N=396 women, N=2 women had missing net worth data, and N=10 had missing covariate data, resulting in a final N=384. An additional participant did not wear the cuff at night, and was excluded from all nighttime BP analyses.

© 2022 Spikes T et al. *JAMA Network Open.*

eResults

Exploratory Analyses

Education, debt stress and depressive symptoms were tested as potential confounders in our primary analyses. However, in order to determine whether associations between net worth and BP outcomes were stronger for women with: 1) lower levels of education (i.e., because women with a higher level of education might ultimately be able to reduce their debt through future opportunities); 2) higher levels of debt stress, or 3) elevated depressive symptoms, we tested interactions between net worth and each of these variables separately in exploratory analyses.

There were no significant interactions observed between net worth and education for DT SBP (p=.27), NT SBP (p=.36), DT DBP (p=.12), NT DBP (p=.16) or sustained hypertension (p=.53). There were also no significant interactions observed between net worth and debt stress for DT SBP (p=.93), NT SBP (p=.70), DT DBP (p=.90), NT DBP (p=.95) or sustained hypertension (p=.66). Similarly, the test of an interaction between net worth and depressive symptoms also yielded non-significant results for DT SBP (p=.42), NT SBP (p=.20), DT DBP (p=.51), NT DBP (p=.11) and sustained hypertension (p=.97).

© 2022 Spikes T et al. *JAMA Network Open.*

eFigure. Net Worth and Nighttime Systolic Blood Pressure in African American Women

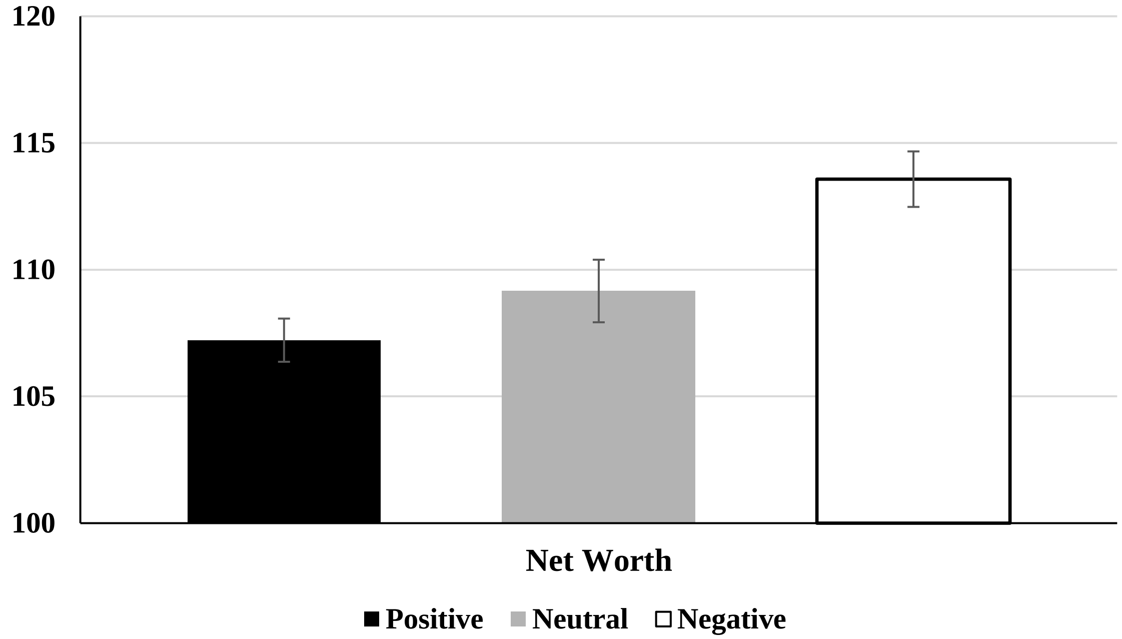

Note: Values are Least-squared means from age adjusted analyses.
P<.0001 for difference between Positive and Negative Net Worth.

© 2022 Spikes T et al. *JAMA Network Open.*

eTable 1. Net Worth and 48-hour Daytime Diastolic Blood Pressure among African American Women not taking antihypertensive medications

	Model 1		*Model 2*		*Model 3*		*Model 4*	
	β (S.E.)	*p-value*	*β (S.E.)*	*p-value*	*β (S.E.)*	*p-value*	*β (S.E.)*	*p-value*
**Net Worth**								
Negative (“in Debt)	3.8 (1.1)	.001	3.8 (1.2)	.001	3.1 (1.2)	.01	3.1 (1.2)	.01
Neutral (“Break Even”)	2.1 (1.2)	.09	2.1 (1.2)	.09	1.8 (1.2)	.15	1.8 (1.2)	.15
Positive (“Left Over”) (ref)	--		--		--		--	
**Education**								
HS or less	0.3 (1.3)	.84	0.2 (1.3)	.87	0.4 (1.3)	.75	0.3 (1.3)	.84
Some College	1.0 (1.3)	.79	1.0 (1.3)	.44	1.0 (1.3)	.45	0.8 (1.3)	.54
College or higher (ref)	--		--		--		--	
**Income**								
$<35K USD	-.57 (1.6)	.72	-.59 (1.6)	.72	-.62 (1.6)	.70	-0.76 (1.6)	.64
$35K-$49,999K	-.79 (1.4)	.57	-.80 (1.4)	.57	-.94 (1.4)	.50	-1.0 (1.4)	.48
$50K-$74,999K	-1.4 (1.3)	.29	-1.4 (1.3)	.29	-1.4 (1.3)	.30	-1.5 (1.3)	.27
≥$75K (ref)	--		--		--		--	

Model 1: in addition to education and income, adjusted for age, marital status, family size; Model 2: Model 1 + BMI and smoking**;** Model 3: Model 2 + debt stress; Model 4: Model 3 + depressive symptoms. *** *p*<.001; ***p*<.01; **p*<.05

© 2022 Spikes T et al. *JAMA Network Open.*

eTable 2: Net Worth and 48-hour Nighttime Diastolic Blood Pressure among African American Women not taking antihypertensive medications

	Model 1		*Model 2*		*Model 3*		*Model 4*	
	β (S.E.)	*p-value*	*β (S.E.)*	*p-value*	*β (S.E.)*	*p-value*	*β (S.E.)*	*p-value*
**Net Worth**								
Negative (“in Debt)	4.2 (1.1)	<.0001	4.0 (1.1)	<.0001	3.6 (1.2)	.003	3.5 (1.2)	.003
Neutral (“Break Even”)	1.4 (1.2)	.22	1.4 (1.2)	.24	1.2 (1.2)	.33	1.2 (1.2)	.32
Positive (“Left Over”) (ref)	--		--		--		--	
**Education**								
HS or less	0.5 (1.3)	.67	-0.9 (1.3)	.50	-0.7 (1.3)	.57	0.9 (1.3)	.49
Some College	0.8 (1.2)	.52	0.7 (1.3)	.59	0.7 (1.3)	.60	0.5 (1.3)	.70
College or higher (ref)	--		--		--		--	
**Income**								
$<35K USD	-0.1 (1.6)	.97	-0.1 (1.6)	.94	-0.2 (1.6)	.92	-0.3 (1.6)	.86
$35K-$49,999K	-0.4 (1.4)	.77	-0.4 (1.4)	.76	-0.5 (1.4)	.71	-0.6 (1.4)	.68
$50K-$74,999K	-1.8 (1.3)	.15	-1.8 (1.3)	.15	-1.8 (1.3)	.16	-1.9 (1.3)	.14
≥$75K (ref)	--		--		--		--	

Model 1: in addition to education and income, adjusted for age, marital status, family size; Model 2: Model 1 + BMI and smoking**;** Model 3: Model 2 + debt stress; Model 4: Model 3 + depressive symptoms. *** *p*<.001; ***p*<.01; **p*<.05

© 2022 Spikes T et al. *JAMA Network Open.*

eTable 3: Net Worth and 48-hour Daytime Systolic Blood Pressure among African American Women, including those on antihypertensive medications

	Model 1		*Model 2*		*Model 3*		*Model 4*	
	β (S.E.)	*p-value*	*β (S.E.)*	*p-value*	*β (S.E.)*	*p-value*	*β (S.E.)*	*p-value*
**Net Worth**								
Negative (“in Debt)	5.5 (1.5)	<.0001	5.3 (1.5)	<.0001	4.6 (1.5)	.003	4.6 (1.5)	.003
Neutral (“Break Even”)	3.3 (1.6)	.04	3.1 (1.5)	.04	2.7 (1.5)	.08	2.7 (1.5)	.08
Positive (“Left Over”) (ref)	--		--		--		--	
**Education**								
HS or less	2.3 (1.7)	.18	2.2 (1.6)	.17	2.3 (1.6)	.15	2.2 (1.6)	.19
Some College	3.0 (1.6)	.06	2.1 (1.6)	.18	2.1 (1.6)	.18	1.9 (1.6)	.23
College or higher (ref)	--		--		--		--	
**Income**								
$<35K USD	0.4 (2.1)	.87	-1.1 (2.1)	.58	-1.2 (2.0)	.56	-1.3 (2.0)	.53
$35K-$49,999K	-1.2 (1.9)	.51	-1.6 (1.8)	.38	-1.8 (1.8)	.33	-1.7 (1.8)	.34
$50K-$74,999K	-0.9 (1.8)	.63	-1.4 (1.7)	.40	-1.4 (1.7)	.39	-1.5 (1.7)	.38
≥$75K (ref)	--		--		--		--	

Model 1: in addition to education and income, adjusted for age, marital status, family size; Model 2: Model 1 + BMI, smoking and anti-hypertensive use; Model 3: Model 2 + debt stress; Model 4: Model 3 + depressive symptoms.

© 2022 Spikes T et al. *JAMA Network Open.*

eTable 4: Net Worth and 48-hour Nighttime Systolic Blood Pressure among African American Women, including those on antihypertensive medications

	Model 1		*Model 2*		*Model 3*		*Model 4*	
	β (S.E.)	*p-value*	*β (S.E.)*	*p-value*	*β (S.E.)*	*p-value*	*β (S.E.)*	*p-value*
**Net Worth**								
Negative (“in Debt)	5.5 (1.4)	<.0001	5.1 (1.4)	<.0001	4.6 (1.4)	.002	4.6 (1.4)	.002
Neutral (“Break Even”)	1.7 (1.5)	.28	1.4 (1.4)	.32	1.1 (1.4)	.43	1.1 (1.4)	.44
Positive (“Left Over”) (ref)	--		--		--		--	
**Education**								
HS or less	1.8 (1.6)	.26	1.4 (1.5)	.35	1.5 (1.5)	.32	1.3 (1.5)	.38
Some College	2.7 (1.5)	.09	1.6 (1.5)	.28	1.6 (1.5)	.28	1.4 (1.5)	.36
College or higher (ref)	--		--		--		--	
**Income**								
$<35K USD	-0.4 (2.0)	.85	-2.0 (1.9)	.30	-2.0 (1.9)	.29	-2.1 (1.9)	.27
$35K-$49,999K	-0.6 (1.8)	.76	-0.9 (1.7)	.58	-1.1 (1.7)	.53	-1.0 (1.7)	.55
$50K-$74,999K	-1.2 (1.7)	.47	-1.7 (1.6)	.28	-1.7 (1.6)	.28	-1.7 (1.6)	.27
≥$75K (ref)	--		--		--		--	

Model 1: in addition to education and income, adjusted for age, marital status, family size; Model 2: Model 1 + BMI, smoking and anti-hypertensive use; Model 3: Model 2 + debt stress; Model 4: Model 3 + depressive symptoms. *** *p*<.001; ***p*<.01; **p*<.05

© 2022 Spikes T et al. *JAMA Network Open.*

eTable 5: Net Worth and 48-hour Daytime Diastolic Blood Pressure among African American Women, including those on antihypertensive medications

	Model 1		*Model 2*		*Model 3*		*Model 4*	
	β (S.E.)	*p-value*	*β (S.E.)*	*p-value*	*β (S.E.)*	*p-value*	*β (S.E.)*	*p-value*
**Net Worth**								
Negative (“in Debt)	3.2 (1.1)	.004	3.4 (1.1)	.002	2.9 (1.1)	.01	2.9 (1.1)	.01
Neutral (“Break Even”)	2.1 (1.2)	.06	2.2 (1.1)	.05	1.9 (1.1)	.09	1.9 (1.1)	.09
Positive (“Left Over”) (ref)	--		--		--		--	
**Education**								
HS or less	1.3 (1.2)	.30	1.4 (1.2)	.25	1.5 (1.2)	.22	1.4 (1.2)	.26
Some College	1.9 (1.2)	.09	1.3 (1.2)	.28	1.3 (1.2)	.28	1.2 (1.2)	.33
College or higher (ref)	--		--		--		--	
**Income**								
$<35K USD	-0.4 (1.5)	.80	-1.3 (1.5)	.39	-1.3 (1.5)	.37	-1.4 (1.5)	.35
$35K-$49,999K	-0.7 (1.4)	.63	-0.9 (1.4)	.48	-1.0 (1.3)	.43	-1.0 (1.3)	.44
$50K-$74,999K	-0.5 (1.3)	.70	-0.9 (1.3)	.47	-0.9 (1.2)	.47	-0.9 (1.2)	.46
≥$75K (ref)	--		--		--		--	

Model 1: in addition to education and income, adjusted for age, marital status, family size; Model 2: Model 1 + BMI, smoking and anti-hypertensive use; Model 3: Model 2 + debt stress; Model 4: Model 3 + depressive symptoms. *** *p*<.001; ***p*<.01; **p*<.05

© 2022 Spikes T et al. *JAMA Network Open.*

eTable 6: Net Worth and 48-hour Nighttime Diastolic Blood Pressure among African American Women, including those on antihypertensive medications

	Model 1		*Model 2*		*Model 3*		*Model 4*	
	β (S.E.)	*p-value*	*β (S.E.)*	*p-value*	*β (S.E.)*	*p-value*	*β (S.E.)*	*p-value*
**Net Worth**								
Negative ("in Debt)	3.7 (1.1)	.001	3.7 (1.1)	.001	3.4 (1.1)	.002	3.4 (1.1)	.003
Neutral ("Break Even")	1.0 (1.1)	.36	1.0 (1.1)	.36	0.9 (1.1)	.44	0.8 (1.1)	.46
Positive ("Left Over") (ref)	--		--		--		--	
**Education**								
HS or less	0.3 (1.2)	.80	0.2 (1.2)	.88	0.2 (1.2)	.85	0.1 (1.2)	.94
Some College	2.0 (1.2)	.09	1.2 (1.1)	.30	1.1 (1.1)	.30	1.0 (1.1)	.37
College or higher (ref)	--		--		--		--	
**Income**								
$<35K USD	-0.1 (1.5)	.94	-1.1 (1.5)	.47	-1.1 (1.5)	.46	-1.1 (1.5)	.44
$35K-$49,999K	-0.3 (1.3)	.83	-0.6 (1.3)	.65	-0.6 (1.3)	.62	-0.6 (1.3)	.64
$50K-$74,999K	-0.9 (1.3)	.48	-1.2 (1.2)	.32	-1.2 (1.2)	.32	-1.2 (1.2)	.31
≥$75K (ref)	--		--		--		--	

Model 1: in addition to education and income, adjusted for age, marital status, family size; Model 2: Model 1 + BMI, smoking and anti-hypertensive use; Model 3: Model 2 + debt stress; Model 4: Model 3 + depressive symptoms. *** *p*<.001; ***p*<.01; **p*<.05

© 2022 Spikes T et al. *JAMA Network Open.*

eTable 7. Net Worth and Sustained Hypertension among African American Women, including those on antihypertensive medications

	Model 1	*Model 2*	*Model 3*	*Model 4*
	OR (95% CI)	*OR (95% CI)*	*OR (95% CI)*	*OR (95% CI)*
**Net Worth**				
Negative (“in Debt”)	2.0 (1.2, 3.5)	2.2 (1.2, 3.8)	1.8 (1.0, 3.4)	1.8 (1.0, 3.4)
Neutral (“Break Even”)	1.5 (0.9, 2.8)	1.6 (0.9, 2.9)	1.5 (0.8, 2.7)	1.5 (0.8, 2.7)
Positive (“Left Over”) (ref)	--	--	--	--
**Education**				
HS or less	1.6 (0.9, 2.9)	1.7 (0.9, 3.2)	1.8 (0.9, 3.3)	1.7 (0.9, 3.3)
Some College	2.0 (1.1, 3.5)	1.8 (1.0, 3.4)	1.8 (1.0, 3.4)	1.8 (0.9, 3.3)
College or higher (ref)	--	--	--	--
**Income**				
$<35K USD	1.2 (0.6, 2.7)	1.0 (0.4, 2.1)	1.0 (0.4, 2.1)	0.9 (0.4, 2.1)
$35K-$49,999K	1.0 (0.5, 2.1)	1.0 (0.5, 2.0)	0.9 (0.4, 1.9)	1.0 (0.5, 2.0)
$50K-$74,999K	1.0 (0.5, 1.9)	0.9 (0.4, 1.7)	0.9 (0.4, 1.7)	0.9 (0.4, 1.7)
≥$75K (ref)	--	--	--	--

Model 1: in addition to education and income, adjusted for age, marital status, family size; Model 2: Model 1 + BMI, smoking and anti-hypertensive use; Model 3: Model 2 + debt stress; Model 4: Model 3 + depressive symptoms.

© 2022 Spikes T et al. *JAMA Network Open.*
